# 肺癌细胞中miR-182启动子甲基化状态研究

**DOI:** 10.3779/j.issn.1009-3419.2015.05.02

**Published:** 2015-05-20

**Authors:** 永文 李, 永林 孙, 凡 任, 颖 李, 明辉 刘, 红雨 刘, 军 陈

**Affiliations:** 1 300052 天津，天津市肺癌研究所 Tianjin Lung Cancer Institute; 2 天津医科大学总医院肺部肿瘤外科 Department of Lung Cancer Surgery, Tianjin Medical University General Hospital, Tianjin 300052, China

**Keywords:** DNA甲基化, miR-182, 肺肿瘤, DNA methylation, miR-182, Lung neoplasms

## Abstract

**背景与目的:**

已有的研究证明MiR-182的异常调控与恶性肿瘤的发生发展密切相关，本研究旨在探讨肺癌细胞中miR-182启动子的甲基化状态对miR-182表达的影响。

**方法:**

荧光定量PCR检测肺癌细胞中miR-182表达水平，甲基化特异性PCR检测各细胞株中miR-182启动子区的甲基化状态，并通过测序进行验证。DNA甲基转移酶抑制剂5’-Aza-dC处理后检测各肺癌细胞株中miR-182表达变化。

**结果:**

MiR-182在不同肺癌细胞株的表达水平不同，其中，在高转移性肺癌细胞株如A549和L9981中相对呈低表达；而在低转移性细胞株95C则相对呈高表达。MSP及测序分析显示多株肺癌细胞株中miR-182启动子区域存在DNA甲基化，其中A549细胞甲基化程度最高。在5'-氮杂-脱氧胞苷酸（5’-Aza-dC）作用下，A549细胞及其他肺癌细胞中miR-182表达水平均明显升高。

**结论:**

在肺癌细胞中miR-182启动子区域存在异常甲基化，miR-182的表达受DNA甲基化的调控。MiR-182的甲基化在肺癌中的作用尚需进一步研究。

## Abstract (en)

**Background and objective:**

It has been proven that the abnormal expression of miR-182 was related to the occurrence and development of tumors. The aim of this study is to explore the relationship between the methylation of miR-182 promoter and its expression in lung cancer cell lines.

**Methods:**

Real-time quantitative PCR and methylation-specific PCR were used to detect the expression level of miR-182 and its promoter methylation status in five lung cancer cell lines (A549, L9981, NL9980, 95C and 95D). DNA sequencing was used to confirm the methylation results.

**Results:**

The level of miR-182 expression significantly differs among these lung cancer cell lines. The highly metastatic human lung cancer cell lines, namely, A549 and L9981, demonstrate a relatively lower expression level of miR-182 compared with the lowly metastatic human lung cancer cell line 95C. Methylation-specific PCR and DNA sequencing assay results indicate that these lung cancer cell lines present different levels of miR-182 promoter methylation, and the highest methylation level is observed in A549 cells. Furthermore, the expression of miR-182 in these cell lines significantly increases when treated with 10 μM 5'-Aza-dC.

**Conclusion:**

DNA methylation occurs in the miR-182 promoter region in lung cancer cell lines. This methylation can regulate the expression level of miR-182. Further study must be conducted to explore the function of miR-182 promoter methylation in lung cancer occurrence and development.

肺癌是呼吸系统最常见的恶性肿瘤，其发病率和死亡率在世界范围内居高不下，特别是在我国大中城市呈现逐年上升的趋势。肺癌的发生发展是一个多步骤的复杂过程，涉及多个基因的异常调控^[1]^。近年来，随着肿瘤研究的深入，表观遗传学异常修饰在肺癌发生过程中的作用越来越受到重视，众多的证据显示，DNA异常甲基化与miRNA的异常调控对肿瘤发生发展起重要的作用^[2]^。

MiRNAs是一类保守的、非编码蛋白的单链小分子，在转录后水平调控靶基因表达，具有广泛的基因调节功能，可调节基因活动各个层面，如生长、分化、凋亡等^[3]^。研究^[4]^证明miRNAs参与生命过程中一系列重要进程，包括早期胚胎发育、细胞增殖、细胞凋亡、细胞死亡以及肿瘤发生、发展和侵袭转移等。据估计，人类中存在1, 000余种miRNAs，约1/3基因表达受miRNAs调控。MiRNAs大多与其靶mRNA的3′非翻译区（untranslated region, UTR）一定程度地互补配对，如果互补配对程度高（大多数植物中），则可导致靶基因mRNA降解；如果互补配对程度低（大多数动物中），则可抑制靶基因mRNA的翻译。目前已研究证实，miRNAs在肿瘤发生发展过程中起着相当重要的作用，而成为近年来研究的热点。如研究发现miRNA-200家族^[5]^、miR-155^[6]^等多种miRNAs参与调控肿瘤的发生发展过程。此外，目前的研究还表明，许多miRNAs基因内部或邻近的CpG岛发生异常甲基化修饰导致的miRNAs异常表达，有可能对肿瘤的发生发展起重要的调控作用。

MiR-182是miR-183家族（包括miR-183、miR-182、miR-96三种miRNA）的成员之一。miR-182位于人染色体7q31-34区，与人*c*-*Met*、*BRAF*等癌基因相邻^[7]^。研究^[8-12]^发现，miR-182是一种肿瘤特异性表达miRNA，其异常表达与肺癌、乳腺癌、肝癌、结直肠癌的发病机制相关。Segura等^[7]^报道miRNA-182可以促进黑色素瘤侵袭转移。他们的实验证实高表达miRNA-182在体内外均可促进黑色素瘤细胞侵袭转移，而下调miR-182可阻止细胞侵袭转移并引发细胞凋亡。进一步研究还证实miR-182通过直接抑制FOXO3和小眼球相关转录因子而促进侵袭转移，而增加FOXO3和小眼畸形相关转录因子（microphthalmia-associated transcription factor, MiTF）的表达，还可抑制miR-182的促侵袭转移效果。Zhang等^[13]^发现，在人肺腺癌细胞株A549中，miR-182通过抑制人的皮肌动蛋白（cortactin, *CTTN*）基因，而抑制细胞的增殖和侵袭。

多项研究表明，肿瘤细胞中miR-182启动子区存在着异常甲基化修饰。如Liu等^[14]^报道在黑色素瘤细胞中miR-182启动子区的DNA存在异常甲基化。Xu等^[15]^也报道，miR-182启动子上游8 kb-10 kb区域存在CpG岛，5’-Aza-dC处理可提高肿瘤细胞中miR-182的表达，从而促进miR-182在肿瘤细胞中的作用。而miR-182在肺癌细胞中的甲基化情况尚未有报道。本研究首先在不同肺癌细胞系中初步研究miR-182启动子调控区的甲基化状态，为进一步阐明miR-182在肺癌发生发展过程中的作用奠定基础。

## 1 材料与方法

### 1.1 主要试剂及仪器

人肺癌细胞系A549（购自美国ATCC）、95C、95D（由军事医学科学院陆应麟教授惠赠）、NL9980和L9981（由周清华教授构建）。以上细胞系由天津市肺癌研究所保存。RMPI 1640和DMEM培养基、胎牛血清及Trizol试剂购自Life Technologies公司（Carlsbad, CA, USA）；实时荧光定量PCR试剂盒、Premix Ex Taq Hotstart Version购自Takara公司（Dalian, China）；反转录试剂盒购自Promega公司（Madison, WI, USA）；Bulge-Loop^TM^ miRNA qRT-PCR Primer Set购自广州锐博生物科技有限公司（Guangzhou, China）；QIAamp DNA Mini Kit和EpiTect Bisulfite Kit购自Qiagen公司（Hilden, Germany）；甲基转移酶抑制剂5’-氮杂-2’-脱氧胞苷（5’-Aza-dC）购自Sigma-Aldrich公司（Kansas, Missouri, USA）；青霉素-链霉素溶液购于碧云天生物技术研究所（Haimen, China）。

### 1.2 细胞培养及DNA甲基转移酶抑制剂5’-Aza-dC处理

A549、95C、95D、NL9980和L9981细胞培养于10 cm培养皿，37 ℃、5%CO_2_饱和湿度的培养箱中，培养基为含10%胎牛血清的RPMI 1640培养基或DMEM培养基。0.25%胰酶-EDTA消化传代，所有实验均采用对数生长期细胞。DNA甲基转移酶抑制剂5’-Aza-dC溶于DMSO溶液中，用10 μmol/L浓度处理6孔板中细胞（每孔2×10^5^细胞），3个复孔，连续处理72 h，每24 h更换新鲜培养液。

### 1.3 MiR-182基因表达的real-time PCR检测

Trizol法常规提取细胞总RNA，根据广州锐博生物科技有限公司Bulge-Loop^TM^ miRNA qRT-PCR Primer Set产品说明书将2 μg RNA进行逆转录，合成cDNA，并置于实时荧光定量PCR仪Applied Biosystems 7900HT Fast Real-Time PCR System instrument and software（Applied Biosystems, USA）进行real-time PCR反应。反应条件：95 ℃ 20 s；40个PCR循环（95 ℃ 10 s；60 ℃ 20 s；72 ℃ 10 s）。以U6作为内参照，A549作为矫正因子，数据采用2^-∆∆CT^法进行分析，ΔCT=CT（miR-182）-CT（U6），ΔΔCT=ΔCT（其他细胞）-ΔCT（A549）。

### 1.4 基因组DNA提取

收集细胞沉淀，按QIAamp DNA Mini Kit说明书步骤进行DNA提取，DNA提取后琼脂糖电泳检测DNA纯度，并用紫外分光光度计进行定量。

### 1.5 MiR-182启动子查找及甲基化特异性PCR（methylation-specific PCR, MSP）引物设计

运用UCSC数据库预测人miR-182启动子序列，提取启动子上游8 kb-10 kb区域。分别利用MethPrimer在线分析网站和Methyl Primer Express v1.0软件分析并预测人miR-182启动子甲基化CpG岛，并通过Methyl Primer Express v1.0软件设计甲基化特异性引物和非甲基化引物。

### 1.6 甲基化特异性PCR（methylation-specific PCR, MSP）检测

基因组DNA亚硫酸盐修饰：每个样本取800 ng为模板进行重亚硫酸盐处理。DNA的重亚硫酸盐修饰按照EpiTect Bisulfite Kit（QINGEN）的说明书，在PCR仪上进行，修饰条件：99 ℃ 5 min，60 ℃ 25 min，99 ℃ 5 min，60 ℃ 85 min，99 ℃ 5 min，60 ℃ 175 min，最后置20 ℃不超过24 h。修饰完成后按照试剂盒附带的纯化试剂说明书对DNA进行纯化，纯化后-20 ℃保存备用。修饰后目的片段PCR扩增和产物的凝胶纯化：基因组DNA重亚硫酸盐处理并纯化后，取1 µL DNA为模板进行扩增。MSP引物：M-forward: 5’-TAGGGGTCGTTCGATTTTAC-3’，M-reverse: 5’-CTACCCCCGACGAATATTACTA-3’，目的条带为103 bp；非甲基化引物：U-forward: 5’-GTTAGGGGTTGTTTGATTTTAT-3’，M-reverse: 5’-CCTACCCCCAACAAATATTACTAT-3’，目的条带为103 bp，使用Takara热启动酶进行扩增。PCR循环条件为：95 ℃预变性3 min，98 ℃ 10 s，48 ℃ 30 s，72 ℃ 30 s，5个循环；95 ℃ 15 s，50 ℃ 30 s，72 ℃ 30 s，5个循环；95 ℃ 15 s，52 ℃ 30 s，72 ℃ 30 s，10个循环；95 ℃ 15 s，54 ℃ 30 s，72 ℃ 30 s，20个循环；72 ℃延伸5 min，4 ℃保存。扩增产物经2%琼脂糖电泳进行鉴定，阴性对照采用双蒸水为模板。上述PCR产物直接交由由北京六合华大基因公司测序，经NCBI Blast判定结果。

### 1.7 统计学方法

应用SPSS 21.0统计软件进行分析，高低转移细胞系中miR-182的表达水平比较、同一细胞系处理组与对照组间的miR-182的表达比较采用*t*检验，*P* < 0.05为差异有统计学意义。

## 2 结果

### 2.1 不同肺癌细胞系中miR-182的表达

采用real-time PCR方法，检测不同肺癌细胞系中miR-182的表达。结果如图 1所示，不同肺癌细胞系中miR-182的表达不同，其中，在人高转移大细胞肺癌细胞L9981的表达明显低于人低转移大细胞肺癌细胞NL9980（*P* < 0.05），在人高转移肺腺癌细胞95D的表达明显低于人低转移肺腺癌细胞95C（*P* < 0.01），而miR-182在高转移细胞系A549中的表达最低。

**1:**
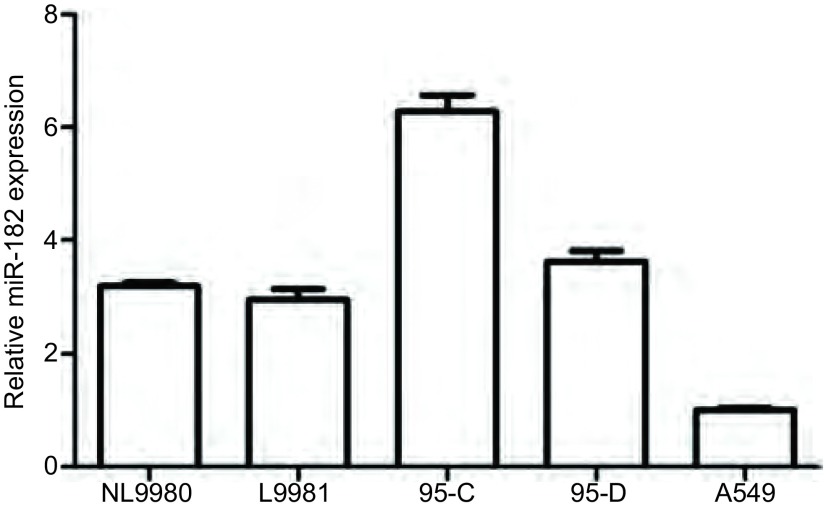
不同肺癌细胞株中miR-182表达水平 The expression level of miR-182 in different lung cancer cell lines by real-time PCR

### 2.2 miR-182启动子区CpG岛的查找及MSP引物设计

应用UCSC数据库预测出人miR-182 DNA启动子序列，并获取启动子上游8 kb-10 kb区域共2, 400 bp，再通过MethPrimer在线分析网站和Methyl Primer Express v1.0软件分析这段序列的CpG岛，采用标准：碱基对>300，GC%>50.0%，观察值/预测值>0.6。结果显示，miR-182 DNA启动子的CpG岛长度1, 989 bp，位于miR-182上游148 bp-2, 138 bp（图 2）。通过Methyl Primer Express v1.0软件设计出7对MSP-PCR引物，并筛选出合适的引物。

**2:**
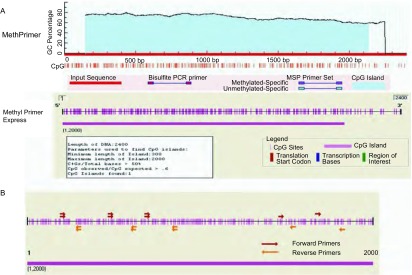
miR-182启动子CpG岛的预测及MSP引物设计。A: MethPrimer数据库和MethyPrimer Express软件预测的miR-182CpG岛；B：MSP引物设计示意图 The prediction of miR-182 promoter CpG island and MSP primers design. A: Prediction of miR-182 CPG island using MethPrimer database and MethyPrimer Express software; B: Schematic diagram of MSP primer design. MSP: methylation-specific PCR

### 2.3 不同肺癌细胞株中miR-182启动子甲基化状态的检测

提取不同肺癌细胞系的DNA，应用重亚硫酸盐处理，未甲基化的胞嘧啶（C）将转变为尿嘧啶（U），而甲基化的胞嘧啶（C）不变。然后将重亚硫酸盐处理后的DNA运用MSP-PCR来检测这些肺癌细胞系中miR-182启动子的甲基化情况。如图 3所示：MSP结果显示5种肺癌细胞系中均存在不同程度的甲基化，其中，A549细胞中miR-182启动子区域甲基化程度最高，其余4株细胞系中miR-182也都存在不同程度的甲基化。PCR产物经DNA测序分析，证实扩增产物为存在甲基化的miR-182启动子区（图 4）。

**3:**
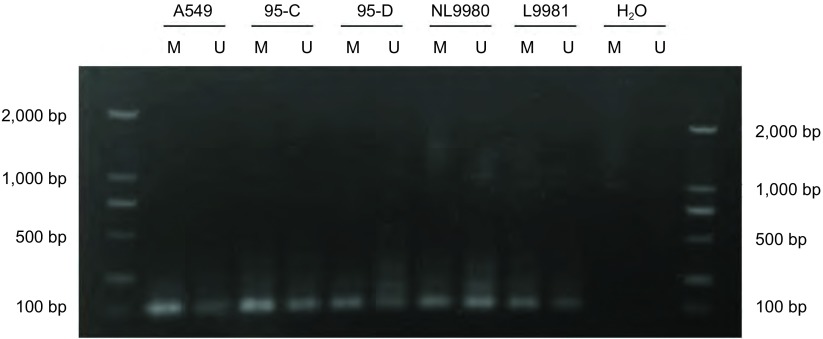
不同肺癌细胞中miR-182启动子区甲基化的MSP检测 MSP assay for miR-182 promoter in different lung cancer cell lines

**4:**
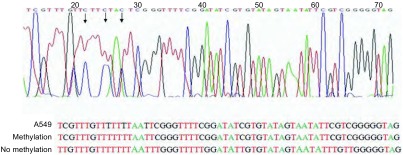
A549细胞中miR-182启动子MSP产物测序分析 The MSP result analysis of miR-182 promoter by DNA sequencing in A549 cells

### 2.4 5'-Aza-dC处理后不同肺癌细胞株中miR-182表达变化

为了进一步探讨DNA甲基化对miR-182表达的影响，我们应用甲基转移酶抑制剂5'-Aza-dC处理细胞72 h后，real-time PCR检测miR-182的表达水平，以DMSO处理作为对照组。结果显示：与对照组相比，在10 μM 5'-Aza-dC处理72 h后，A549、95C、95D、L9981和NL9980肺癌细胞株中miR-182的表达均提高（分别为*P* < 0.001、*P* < 0.01、*P* < 0.01、*P* < 0.05和*P* < 0.001），其中A549细胞中miR-182的表达提高了406倍，最为明显（图 5）。这些结果表明，启动子区的DNA甲基化可能抑制miR-182的表达，而DNA甲基转移酶抑制剂5'-Aza-dC处理则能提高miR-182的表达。

**5:**
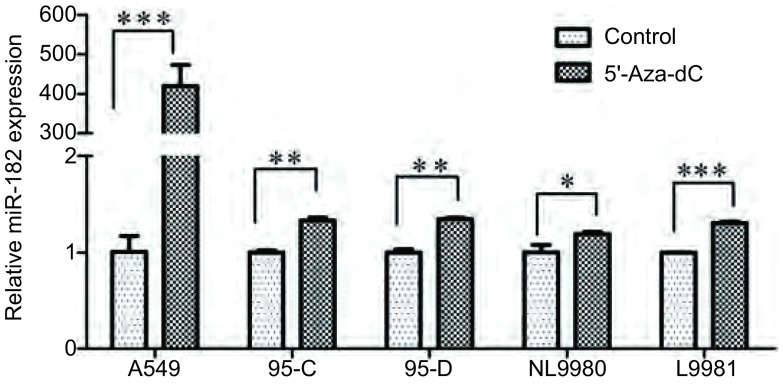
5’-Aza-dC影响肺癌细胞中miR-182表达 The analysis of miR-182 expression by real-time PCR in human lung cancer cell lines after 5'-Aza-dC treatment. ^*^P < 0.05, ^**^P < 0.01, ^***^P < 0.001

## 3 讨论

DNA甲基化是指生物体在DNA甲基转移酶（DNA methyltransferase, DNMT）的催化下，以S-腺苷甲硫氨酸（S-adenosyl methionine, SAM）为甲基供体，将甲基转移到特定的碱基上的过程，是哺乳动物遗传外修饰的重要的调控方式。研究^[16]^表明，DNA异常甲基化与肿瘤的发生、发展、细胞癌变有着密切的联系。启动子区域CpG岛的高甲基化是抑瘤基因沉默、促凋亡基因失活的主要方式之一。因此，改变DNA甲基化状态，可以诱导因甲基化失活的基因重新表达^[17]^。5’-Aza-dC是一种DNA甲基转移酶1（DNA methyltransferas 1, DNMT1）的抑制剂，通过与DNA甲基转移酶的共价结合，抑制DNA甲基转移酶的活性，从而实现去甲基化功能。应用5-Aza-dC可以使部分沉默的重要基因去甲基化而再激活，恢复其正常功能。因此，5’-Aza-dC已被用于Ⅲ期临床试验，用于早期非小细胞肺癌及晚期三阴乳腺癌的临床治疗。

我们的研究结果证实，miR-182启动子区在肺癌细胞株中普遍存在异常的甲基化，特别是在高转移性肺癌细胞株A549中，存在高甲基化状态，经DNA甲基转移酶抑制剂5’-Aza-dC处理后，A549及其它肺癌细胞中miR-182的表达水平均提高，MSP及基因测序分析亦进一步证实这些细胞中存在异常甲基化修饰。因此，在肺癌细胞中，miR-182的表达受异常甲基化调控。

MiR-182在肿瘤的发生发展中起着重要作用，比如，在黑色素瘤细胞中，miR-182可能通过抑制FOXO3和MiTF，促进肿瘤细胞的侵袭和转移^[5]^，而我们的研究发现，在高度转移性的肺癌细胞株A549中，miR-182为高度甲基化，从而提示在肺癌细胞中miR-182的功能可能有别于黑色素瘤细胞，可能与抑制肺癌侵袭和转移有关，这与Zhang等^[13]^的研究报道相一致。这些现象提示miR-182在不同肿瘤或细胞类型中由于其靶基因不同，其作用也不尽相同。miR-182在肺癌细胞株中的异常表达受到DNA甲基化的调控，其作用机制还有待进一步研究。
